# The adding value of contrast-enhanced CT radiomics: Differentiating tuberculosis from non-tuberculous infectious lesions presenting as solid pulmonary nodules or masses

**DOI:** 10.3389/fpubh.2022.1018527

**Published:** 2022-10-04

**Authors:** Wenjing Zhao, Ziqi Xiong, Di Tian, Kunpeng Wang, Min Zhao, Xiwei Lu, Dongxue Qin, Zhiyong Li

**Affiliations:** ^1^Department of Radiology, The First Affiliated Hospital of Dalian Medical University, Dalian, China; ^2^Department of Radiology, Dalian Public Health Clinical Center, Dalian, China; ^3^GE Healthcare, Beijing, China; ^4^Department of Tuberculosis, Dalian Public Health Clinical Center, Dalian, China; ^5^Department of Radiology, The Second Affiliated Hospital of Dalian Medical University, Dalian, China

**Keywords:** pulmonary tuberculosis, solid pulmonary nodules, radiomics, contrast-enhanced, computed tomography

## Abstract

**Purpose:**

To compare the value of contrast-enhanced CT (CECT) and non-contrast-enhanced CT (NCECT) radiomics models in differentiating tuberculosis (TB) from non-tuberculous infectious lesions (NTIL) presenting as solid pulmonary nodules or masses, and develop a combine radiomics model (RM).

**Materials and methods:**

This study was a retrospective analysis of 101 lesions in 95 patients, including 49 lesions (from 45 patients) in the TB group and 52 lesions (from 50 patients) in the NTIL group. Lesions were randomly divided into training and test sets in the ratio of 7:3. Conventional imaging features were used to construct a conventional imaging model (IM). Radiomics features screening and NCECT or CECT RM construction were carried out by correlation analysis and gradient boosting decision tree, and logistic regression. Finally, conventional IM, NCECT RM, and CECT RM were used for combine RM construction. Additionally, we recruited three radiologists for independent diagnosis. The differential diagnostic performance of each model was assessed using the areas under the receiver operating characteristic curve (AUCs).

**Results:**

The CECT RM (training AUC, 0.874; test AUC, 0.796) outperformed the conventional IM (training AUC, 0.792; test AUC, 0.708), the NCECT RM (training AUC, 0.835; test AUC, 0.704), and three radiologists. The diagnostic efficacy of the combine RM (training AUC, 0.922; test AUC, 0.833) was best in the training and test sets.

**Conclusions:**

The diagnostic efficacy of the CECT RM was superior to that of the NCECT RM in identifying TB from NTIL presenting as solid pulmonary nodules or masses. The combine RM had the best performance and may outperform expert radiologists.

## Introduction

In 2020, approximately 9.9 million people worldwide fell ill with tuberculosis (TB), equivalent to 127 cases per 100,000 population. The majority of these patients are located in the WHO regions of South-East Asia (43%), Africa (25%) and the Western Pacific (18%) (1). TB has become a major cause of death from infectious diseases (2), most commonly in the lungs. Moreover, as the stigma is often distressing, TB takes a serious toll on the mental health of patients (3). The elimination of TB has become a global goal, and the key to achieving it is accurate diagnosis and effective treatment (4). However, the non-specific imaging presentation of TB imposes a significant clinical burden, with TB presenting as solid pulmonary nodules or masses in approximately 6–9% of patients (5, 6). Non-tuberculous infectious lesions (NTIL) in the lungs also have solid pulmonary nodules or masses as their manifestation, such as fungal infection and organized pneumonia. It is hard for radiologists to distinguish between TB and NTIL presenting as solid pulmonary nodules or masses by naked eye and experience. But their treatments are very different. NTIL patients are often treated with antimicrobials, hormones, or surgical resection, whereas TB patients require antituberculous drugs (7, 8). Once TB patients are misdiagnosed, they cannot receive timely and effective treatment, and the more serious consequence is the spread of TB.

Mycobacterium culture is the gold standard for the detection of Mycobacterium TB in clinical samples, but this method is time-consuming (9). Among the sample collection methods, spontaneous sputum is often unsatisfactory, and sputum induction, alveolar lavage, and bronchial washing are harmful to the patients (10, 11). CT-guided lung aspiration biopsy in combination with Xpert MTB/RIF Ultra had high sensitivity for the differential diagnosis of pulmonary TB, lung cancer, and chronic infections (12). However, this method is highly invasive to patients and the procedure is tedious. Compared with these methods, CT imaging diagnosis has the superiority of convenience, rapidness, and non-invasiveness. However, a stable and reliable method is needed to help radiologists improve the diagnostic performance of distinguishing TB from NTIL presenting as solid pulmonary nodules or masses.

The concept of radiomics was first introduced by the Dutch scholar Philippe Lambin in 2012, which refers to the automatic high-throughput extraction of a large number of quantitative features from images (13). Currently, the development of radiomics research has advanced rapidly, and its value in guiding clinical decision-making is increasingly appreciated (14, 15). The majority of studies are based on non-contrast-enhanced CT (NCECT) images. However, some researchers have begun to explore the value of contrast-enhanced CT (CECT) images. CECT images often reflect the blood supply to the lesion, and enhancement attenuation helped distinguish between benign and malignant pulmonary nodules (16, 17). In terms of radiomics, CECT combined with texture analysis had a good diagnostic value for distinguishing pulmonary sclerosing pneumocytoma and atypical peripheral lung cancer, with a sensitivity and specificity of 0.82 and 0.87, respectively (18). The study of Liu et al. (19) showed that texture analysis of CECT could be used to evaluate the pathological grade of lung adenocarcinoma. Additionally, Gao et al. (20) proposed that CECT was more useful than NCECT in the radiomics differentiation of lung preinvasive lesions, minimally invasive adenocarcinomas, and invasive adenocarcinomas.

Previous studies have shown the advantages of CECT-based radiomics for the differential diagnosis of certain chest images. Therefore, the main purpose of this study was to investigate whether CECT radiomics had additional value in differentiating between TB and NTIL presenting as solid pulmonary nodules or masses and to establish the combine radiomics model (RM).

## Materials and methods

### Patients

This study was approved and exempted the informed consent of patients by the ethics committees of the three hospitals.

Inclusion criteria: (1) TB was confirmed by sputum testing or pathology combined with DNA testing, clinical symptoms, medical history, and tuberculin testing, and NTIL was confirmed by pathology or effective anti-inflammatory therapy; (2) lesions located in the peri-pulmonary field; (3) CECT images with layer thickness ≤ 2.5 mm on initial examination; (4) lesions presenting as solid nodules or masses without internal calcification; (5) patients with no history of lung surgery, radiotherapy or chemotherapy. According to the inclusion criteria, 73 patients from the First Affiliated Hospital of Dalian Medical University (Center 1) from January 2010 to February 2022, 25 patients from the Second Affiliated Hospital of Dalian Medical University (Center 2) from April 2019 to February 2022, and 15 patients from Dalian Public Health Clinical Center (Center 3) from December 2017 to July 2021 were collected retrospectively. The exclusion criteria were as follows: (1) Artifacts in CT images had an impact on evaluation. (2) Patients did not have NCECT images with slice thickness ≤ 2.5 mm. Eighteen patients were excluded according to the exclusion criteria.

**Figure 1:**
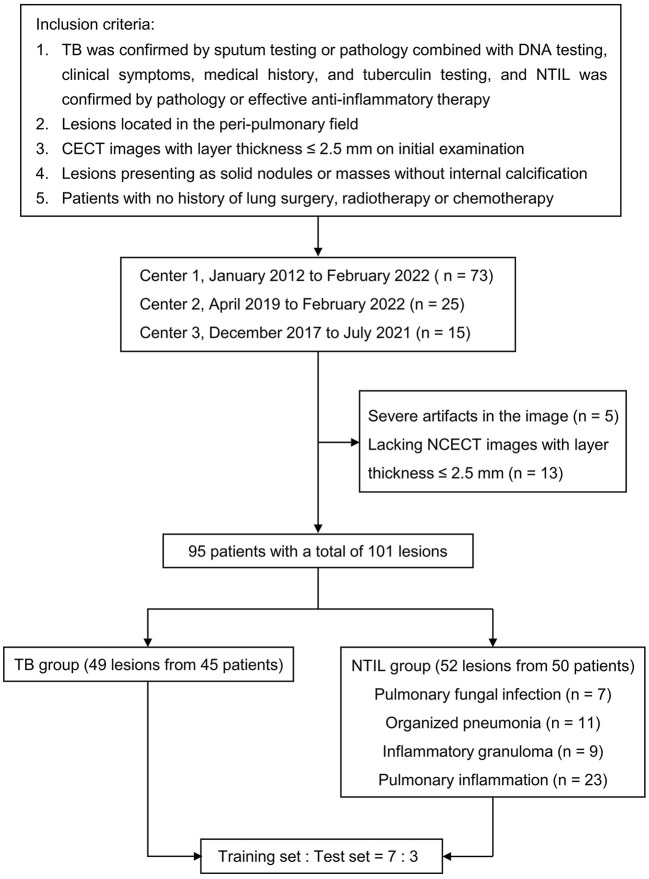
Flowchart of study population. TB, tuberculosis; NTIL, non-tuberculous infectious lesions; NCECT, non-contrast enhanced CT; CECT, contrast-enhanced CT.

The patient selection process is shown in Figure 1. Finally, 95 patients with a total of 101 lesions were included. Among TB patients, 11 cases were confirmed by sputum examination, and 34 cases were confirmed by pathology combined with DNA testing, clinical symptoms, medical history, and tuberculin testing. All of the NTIL patients were pathologically confirmed, except for five pulmonary inflammation patients who were confirmed by effective anti-inflammatory therapy. Details on the selection of lesions for each patient are provided in Supplementary Figure S1. All of the lesions were randomly divided into the training and test sets according to the ratio of 7:3.

### Clinical data

Two demographic characteristics (gender and age), two clinical characteristics (smoking status and clinical symptoms), and five hematological examination indices (white blood cells, neutrophils, lymphocytes, monocytes, and glucose) were recorded at the time of patient admission.

### CT examination acquisition

Chest CT scans were performed using multi-slice spiral CT (16 or more) from three companies (General Electric, American; Siemens, Germany; Philips, Netherlands). The patient was routinely scanned from the tip of the lung to the base under inspiration. Scanning parameters: tube voltage, 120–140 kVp; tube current, 140–630 mA or automatic adjustment; matrix, 512 × 512; reconstruction thickness, 1–2.5 mm; reconstruction interval, 1–2.5 mm. CECT scans were performed using a non-iodine ion contrast agent, with an injection rate of 2.5–3.0 mL/s, and scanned at 55–60 s after contrast injection.

### Image analysis

**Figure 2:**
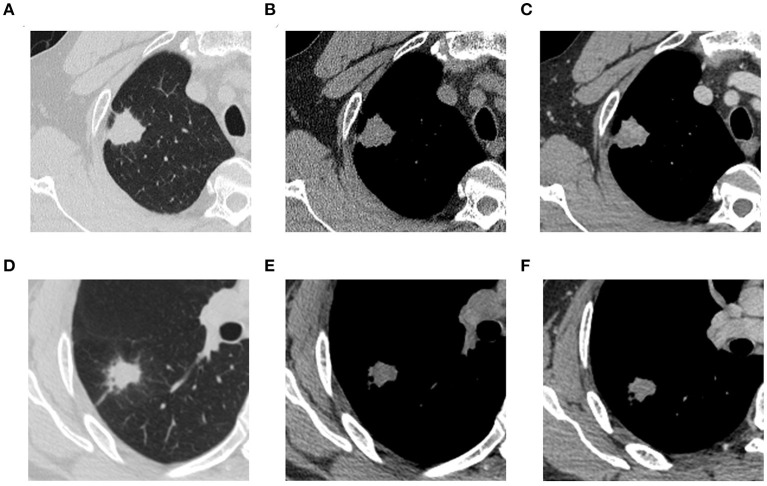
**(A)** (NCECT, lung window), **(B)** (NCECT, mediastinal window), and **(C)** (CECT, mediastinal window) are thoracic CT images of a 53-year-old male patient with pathologically confirmed TB. **(D)** (NCECT, lung window), **(E)** (NCECT, mediastinal window), and **(F)** (CECT, mediastinal window) are thoracic CT images of a 41-year-old male patient with pathologically confirmed organized pneumonia. TB, tuberculosis; NCECT, non-contrast enhanced CT; CECT, contrast-enhanced CT.

CT imaging analysis was done jointly by two thoracic diagnostic radiologists (A and B) without knowing the type of lesions, and different opinions were discussed and agreed upon. Each lesion was observed in the lung window (window level, −700 HU; window width, 1400 HU), mediastinal window (window level, 40 HU; window width, 400 HU), axial, sagittal, and coronal views. The conventional imaging features of each lesion were analyzed, including (1) semantic features: lobulation, spiculation, cavity, pleural traction, and location; and (2) quantitative features: axial position maximum diameter. The apical and posterior segments of the upper lobe (S1 and S2), as well as the superior segment of the lower lobe (S6), are TB predilection sites (21). Therefore, the location was dichotomized, with one category being located in S1 or S2 or S6, and the other category being other locations. Figure 2 shows TB and NTIL lesions.

### Image segmentation and feature extraction

The original images were imported into the open source software 3D slicer (version 4.11.20200930, http://slicer.org/) by radiologist A. The regions of interest (ROIs) were outlined layer by layer on the entire lesion from NCECT and CECT images respectively under a mediastinal window (window width, 400 HU; window level, 40 HU) and the cavity areas were removed. Radiologist A blinded the pathological diagnosis of the lesions.

Adjusting the voxel size to 1 mm × 1 mm × 1 mm, the original images were resampled to automatically extract respective radiomics features in the NCECT and CECT, including first-order statistics, shape-based, gray-level co-occurrence matrix (GLCM), gray-level dependence matrix (GLDM), gray-level run length matrix (GLRLM), gray-level size zone matrix (GLSZM), and neighborhood gray-tone difference matrix (NGTDM).

### Consistency assessment

Assessment of intra- and inter-group agreement was performed by randomly selecting 32 lesions from 101 lesions, and independent measurements were completed by radiologist A and radiologist B with unknown final pathological results. And independent measurements of data were completed again by radiologist A 1 month after the first measurement. Inter- and intra- class correlation coefficients (ICCs) were used to assess the inter- and intra- observer agreement of feature extraction, with ICC > 0.75 indicating good to excellent agreement (Values < 0.5 are indicative of poor reliability, values between 0.5 and 0.75 indicate moderate reliability, values between 0.75 and 0.9 indicate good reliability, and values >0.90 indicate excellent reliability) (22).

### Feature selection and modeling

Firstly, the radiomics feature data of the training and test sets were normalized respectively. Secondly, the radiomics features were screened in the training set using correlation analysis (If the average correlation coefficient of a feature with other features exceeds 0.9, this feature will be rejected.) and gradient boosting decision tree (GBDT). Differences in conventional imaging characteristics of TB and NTIL groups were compared by univariate analysis. Finally, logistic regression was used to establish four models, i.e., conventional imaging model (IM), NCECT RM, CECT RM, and combine RM. The combine RM was formed by the combination of conventional IM, NCECT RM, and CECT RM. The radscore was calculated for each patient *via* a linear combination of selected features that were weighted by their respective coefficients.

### Model evaluation and comparison

The receiver operating characteristic (ROC) curves were performed to evaluate the discriminative performance of the models. The areas under the ROC curves (AUCs), sensitivities, specificities, and accuracies were calculated. The calibration curves were plotted, and the fits of the models were estimated by the Hosmer-Lemeshow (H-L) test. The decision curve analysis (DCA) was performed to estimate the clinical utility of the models.

We invited a senior radiologist C (with 25 years of diagnostic chest imaging experience), a Mid-level radiologist D (with 12 years of diagnostic chest imaging experience), and a junior radiologist E (with 4 years of diagnostic chest imaging experience) to diagnose all lesions independently. Their diagnostic ability in identifying TB from NTIL presenting as solid pulmonary nodules or masses was assessed. The value of RMs was further clarified by comparison with radiologists.

### Statistical analysis

All statistical analyses of the data were performed using R (version 4.1.1). Categorical variables were expressed as the “number of cases (percentage)” and the chi-square test or Fisher's exact test was used to evaluate differences between the TB and NTIL groups. When continuous variables conformed to the normal distribution, they were expressed as mean ± standard deviation and *t*-test was used; when they did not conform to the normal distribution, they were expressed as median (first quartile, third quartile) and Mann-Whitney U test was used. A two-tailed *p*-value < 0.05 indicated statistical significance.

## Results

### Analysis of clinical data and conventional imaging features

There were 45 patients (49 lesions) in the TB group (29 males and 16 females; mean age 50.96 ± 10.94 years), and 50 patients (52 lesions) in the NTIL group (32 males and 18 females; mean age 55.38 ± 10.59 years). The patients in the NTIL group included 7 cases of pulmonary fungal infection, 11 cases of organized pneumonia, 9 cases of inflammatory granuloma, and 23 cases of pulmonary inflammation.

The clinical data of patients in the training and test sets are shown in Table 1. In the training set, the differences in all indicators were not significant (*P* > 0.05). In the test set, more patients in the TB group had a history of smoking than in the NTIL group (*P* = 0.025).

**Table 1:** Clinical information of the patients.

**Variables**	**Training set**			**Test set**		
	**TB**	**NTIL**	***P*** **-value**	**TB**	**NTIL**	***P*** **-value**
No. of patients	32	35		13	15	
Age(y)	53.00 (43.50, 59.00)	58.00 (50.00, 66.00)	0.055	50.54 ± 7.86	53.20 ± 9.73	0.438
Gender			0.813			0.778
Men	21 (65.63%)	22 (62.86%)		8 (61.54%)	10 (66.67%)	
Women	11 (34.37%)	13 (37.14%)		5 (38.46%)	5 (33.33%)	
Smoking status			0.122			0.025
Never smoked	17 (53.13%)	25 (71.43%)		5 (38.46%)	12 (80.00%)	
Ex- or current smoker	15 (46.87%)	10 (28.57%)		8 (61.54%)	3 (20.00%)	
Clinical symptoms			0.853			0.978
No	13 (40.63%)	15 (42.86%)		7 (53.85%)	8 (53.33%)	
Yes	19 (59.37%)	20 (57.14%)		6 (46.15%)	7 (46.67%)	
WBC	5.56 (4.52, 6.61)	5.95 (4.65, 7.39)	0.173	5.98 (5.43, 7.31)	5.95 (4.77, 7.73)	0.945
NEUT	3.24 (2.46, 4.07)	3.20 (2.74, 4.49)	0.581	3.79 (3.14, 4.03)	3.61 (2.64, 5.11)	0.945
LYMPH	1,63 (1.28, 1.92)	1.83 (1.51, 2.13)	0.107	1.97 ± 0.45	1.93 ± 0.77	0.890
MONO	0.40 ± 0.14	0.44 ± 0.18	0.425	0.45 ± 0.16	0.44 ± 0.19	0.917
Glu	6.34 (5.10, 8.71)	5.32 (4.91, 5.99)	0.059	5.35 (4.65, 6.20)	5.68 (4.97, 6.21)	0.519

TB, tuberculosis; NTIL, non-tuberculous infectious lesions; WBC, white blood cell; NEUT, neutrophil; LYMPH, lymphocyte; MONO, monocyte; Glu, glucose.

The comparison of conventional imaging features between two groups in the training and test sets is shown in Table 2. After univariate analysis, the difference in cavity between the two groups was statistically significant in the training set (*P* = 0.001). In the test set, the differences in all features were not significant (*P* > 0.05).

**Table 2:** The comparison of conventional imaging features between two groups.

**Features**	**Training set**			**Test set**		
	**TB**	**NTIL**	***P*** **-value**	**TB**	**NTIL**	***P*** **-value**
No. of lesions	34	36		15	16	
AMD (cm)	2.45 ± 0.88	2.27 ± 0.93	0.415	2.41 ± 1.41	2.45 ± 1.30	0.926
Lobulation			0.858			0.685
No	6 (17.65%)	8 (22.22%)		4 (26.67%)	3 (18.75%)	
Yes	28 (82.35%)	28 (77.78%)		11 (73.33%)	13 (81.25%)	
Spiculation			1.000			1.000
No	9 (26.47%)	10 (27.78%)		4 (26.67%)	5 (31.25%)	
Yes	25 (73.53%)	26 (72.22%)		11 (73.33%)	11 (68.75%)	
Cavity			0.001			0.172
No	23 (67.65%)	36 (100.00%)		11 (73.33%)	15 (93.75%)	
Yes	11 (32.35%)	0 (0.00%)		4 (26.67%)	1 (6.25%)	
Pleural traction			0.676			0.333
No	8 (23.53%)	6 (16.67%)		3 (20.00%)	1 (6.25%)	
Yes	26 (76.47%)	30 (83.33%)		12 (80.00%)	15 (93.75%)	
Location			0.193			0.113
S1/S2/S6	24 (70.59%)	20 (55.56%)		13 (86.67%)	9 (56.25%)	
Other	10 (29.41%)	16 (44.44%)		2 (13.33%)	7 (43.75%)	

TB, tuberculosis; NTIL, non-tuberculous infectious lesions; AMD, Axis position maximum diameter; S1, apical segment of upper lobe; S2, posterior segment of upper lobe; S6, superior segment of lower lobe.

### Radiomics feature screening

The radiomics feature selection processes were the same for both the NCECT and CECT RMs. A total of 107 radiomics features were extracted. First, 17 features with ICC < 0.75 were excluded. The consistency assessment showed the ranges of the inter- and intra-class correlation coefficient values for the remaining 90 radiomics features were 0.765–0.994 and 0.853–1.000, respectively. This indicated good to excellent inter- and intra-observer agreement for the 90 radiomics features. Then, after filtering by correlation analysis, 28 NCECT radiomics features and 29 CECT radiomics features were retained. Finally, the GBDT algorithm selected 11 NCECT radiomics features and 10 CECT radiomics features from them as the best features.

### Model construction and comparison

All conventional imaging features were used to establish the conventional IM. The modeling formulas of the NCECT and CECT RMs are shown in Supplementary Figure S2. Conventional IM, NCECT RM, and CECT RM were used for combine RM construction.

**Figure 3:**
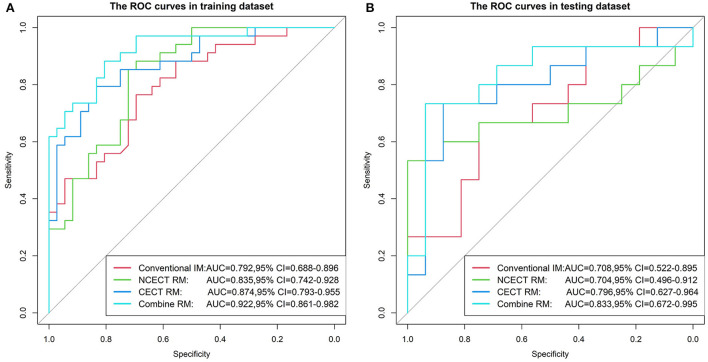
The ROC curves for four models in the training set **(A)** and the test set **(B)**. The AUC of the CECT RM was greater than that of the NCECT RM and the conventional IM. The combine RM achieved the best performance, with AUC of 0.922 and 0.833. ROC, receiver operating characteristic; AUC, area under the ROC curve; NCECT, non-contrast enhanced CT; CECT, contrast-enhanced CT; RM, radiomics model; IM, imaging model.

In the training set, the AUCs of NCECT RM and CECT RM were higher than that of the conventional IM (Table 3; Figure 3). Compared with NCECT RM, the AUCs and accuracies of CECT RM were further improved in the training and test sets (Table 3; Figure 3).

**Table 3:** Diagnostic efficiency of four models and three radiologists.

	**Training set**				**Test set**			
	**AUC (95% CI)**	**Sensitivity**	**Specificity**	**Accuracy**	**AUC (95% CI)**	**Sensitivity**	**Specificity**	**Accuracy**
Conventional IM	0.792 (0.688, 0.896)	0.529	0.833	0.686	0.708 (0.522, 0.895)	0.533	0.750	0.645
NCECT RM	0.835 (0.742, 0.928)	0.853	0.694	0.771	0.704 (0.496, 0.912)	0.600	0.750	0.677
CECT RM	0.874 (0.793, 0.955)	0.765	0.833	0.800	0.796 (0.627, 0.964)	0.800	0.688	0.742
Combine RM	0.922 (0.861, 0.982)	0.824	0.833	0.829	0.833 (0.672, 0.995)	0.733	0.812	0.774
Senior radiologist	0.708 (0.584, 0.833)	0.559	0.806	0.686	0.738 (0.555, 0.920)	0.600	0.875	0.742
Mid-level radiologist	0.627 (0.495, 0.759)	0.588	0.667	0.629	0.417 (0.213, 0.620)	0.333	0.500	0.419
Junior radiologist	0.772 (0.658, 0.887)	0.765	0.778	0.814	0.579 (0.375, 0.783)	0.533	0.625	0.581

AUC, area under the curve; CI, confidence interval; CECT, contrast-enhanced CT; NCECT, non-contrast-enhanced CT; IM, imaging model; RM, radiomics model.

In this study, the best performance was achieved with the combine RM in the training and test sets, with AUCs of 0.922 (95% CI: 0.861, 0.982) and 0.833 (95% CI: 0.672, 0.995), accuracies of 0.829 and 0.774 (Table 3; Figure 3).

**Figure 4:**
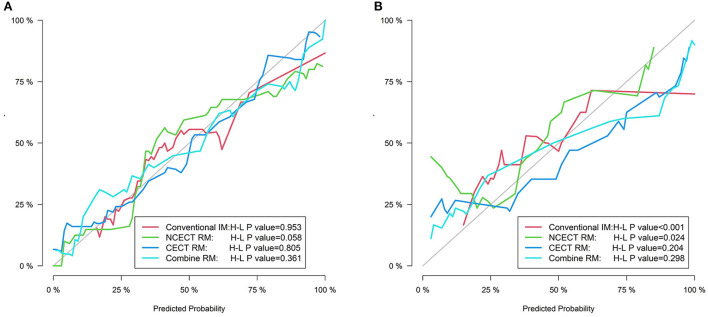
The calibration curves for four models in the training set **(A)** and the test set **(B)**. The gray line represents the ideal prediction effect, and The colored lines represent the prediction effect of each model. The closer the colored line is to the gray line indicates a better fit of the model.

In the training set, the H-L test showed no significant difference between the four models and the ideal model (all *P* > 0.05, Figure 4).

In the training set and the test set, the AUCs and accuracy of the CECT RM and the combine RM were higher than those of the senior, mid-level, and junior radiologists (Table 3).

### Clinical usefulness

**Figure 5:**
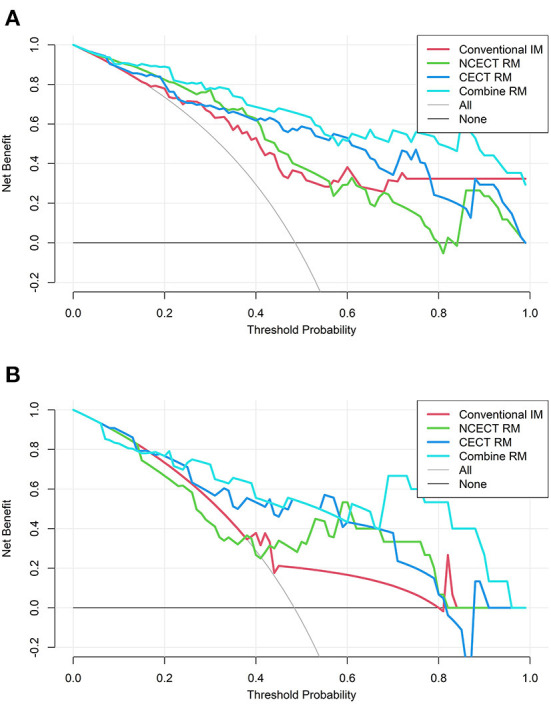
The decision curves for four models in the training set **(A)** and the test set **(B)**. The vertical coordinate represents the net benefit and the horizontal coordinate represents the probability thresholds. None means that all patients were diagnosed with NTIL, no intervention was performed, and the net benefit was 0. All means that all patients were diagnosed with TB, and all patients received intervention, and the net benefit is a backslash with a negative slope. The farther the model curve is from these two extreme curves, the more valuable the model is. TB, tuberculosis; NTIL, non-tuberculous infectious lesions; RM, radiomics model.

The decision curves for each model in the training set and test set are shown in Figure 5. CECT RM had a higher net benefit than NCECT RM over most probability threshold ranges. The combine RM had the highest overall net benefit.

## Discussion

In this study, we used RMs to identify TB and NTIL presenting as solid pulmonary nodules or masses and compared the value of CECT and NCECT. The diagnostic efficacy of RMs based on either NCECT or CECT was improved compared with the conventional IM, more so in the CECT RM. And the combine RM we finally built had the highest differential diagnostic value (training AUC, 0.922; test AUC, 0.833).

Our results showed that cavity was the only conventional imaging feature that differed significantly between the two groups in the training set, and it was more common in TB lesions, accounting for 32.35%. An analysis of the imaging presentation of pulmonary granulomatous lesions also mentioned that cavity was highly suggestive of Mycobacterium tuberculosis infection (23). The proportion of the cavity in their TB lesions was higher than in this study, reaching 57.1%. Moreover, the presence of the cavity may indicate a higher risk of acid-fast bacilli positivity as well as being highly infectious (24).

Given that there was only cavity with the discrepancy, we decided to use all conventional imaging features for modeling to avoid missing important information. However, even though conventional IM integrated all conventional imaging features, its diagnostic power is unsatisfactory (training AUC, 0.792; test AUC, 0.708).

Essentially, CT images provide much more information than the macroscopic appearances we can see with the naked eye. Radiomics then provides us with a large number of quantitative features that reflect the microstructure of the lesion, mainly including intensity histogram, shape-based features, and texture features, and the extraction process of these features is automatic (13, 25). The first-order features reduce the 3D data of the lesion to a single histogram, reflecting the intensity distribution within the ROI. Shape-based features are mainly used to describe the geometry of the lesion. In general, texture features can indirectly reflect the heterogeneity of the tumor (13, 26). In this study, the images are resampled to achieve voxel isotropy, and voxel size resampling can greatly improve the proportion of robust features (27).

Wang et al.'s RM based on NCECT to identify TB and community-acquired pneumonia in children was also quite effective, the AUC reached 0.837 (28). In addition, the radiomics signature established by Feng B et al. (29) based on NCECT could discriminate between TB and adenocarcinoma presenting as solid pulmonary nodules, with good performance in the internal validation set (AUC, 0.890) and external validation set (AUC, 0.874). The aforementioned studies have shown that NCECT-based radiomics can help in the differential diagnosis of TB. And the result of this study was similar. Our NCECT RM (AUC, 0.835) performed well in differentiating between TB and NTIL presenting as solid pulmonary nodules or masses and showed an improvement compared with the conventional IM.

CECT scan is a commonly used auxiliary examination method in clinical diagnosis and treatment. The results of several studies have shown that the radiomics characteristics of tumors are altered after the uptake of contrast agents, particularly first-order histogram features and textural features (30–32). Several studies compared CECT-based radiomics with NCECT-based radiomics and came to different conclusions. Texture analysis of iodine-enhanced images (90 s after contrast injection) produced by dual-energy CT improved the AUC from 0.888 to 0.959 in distinguishing invasive adenocarcinoma from non-invasive or minimally invasive adenocarcinoma compared with virtual non-contrast imaging (33). E et al. (34) compared the value of three CT scan phase RMs in differentiating lung adenocarcinoma and squamous cell carcinoma. The results indicated that there was no significant difference among non-enhanced, arterial, and venous phases. The radiomics signature based on CECT established by He et al. (31) is inferior to NCECT in the identification of benign and malignant solid pulmonary nodules. They chose CECT images with a 25 s delayed scan after contrast injection. In this study, the diagnostic efficacy of the CECT RM (training AUC, 0.874; test AUC, 0.796) was superior to that of the NCECT RM (training AUC, 0.835; test AUC, 0.704). Compared with NCECT radiomics, whether CECT radiomics can bring more value may be related to the types of classification tasks, the pathological structure of lesions, and the scanning phase. This deserves further study.

The AUC (training 0.922; test 0.833) and accuracy (training 0.829; test 0.774) of the combine RM were the highest among all models, and all indicators were relatively balanced in the training and test sets. This study also found that radiologists were not able to distinguish well between TB and NTIL presenting as solid pulmonary nodules or masses, even for experienced radiologists (training AUC, 0.708; test AUC, 0.738). Moreover, there is a great deal of subjectivity and fortuity in the diagnosis of radiologists with different qualifications. Computer-aided diagnosis has become an irreversible trend in clinical work, and artificial intelligence has great prospects in the field of thoracic radiology (35). The combine RM established in this study is of great significance for the diagnosis of TB, especially in areas of high TB prevalence such as the WHO regions of South-East Asia, Africa, and the Western Pacific. First, when doctors encounter solid nodules or masses that are difficult to diagnose in clinical work, they should actively recommend patients undergo enhanced CT examinations. The combine RM can then serve as a reliable reference tool to assist radiologists in differentiating between TB and NTIL if the patient has symptoms of fever and cough, or has a history of exposure to Mycobacterium tuberculosis, or has a long course of disease. However, before the combine RM can be used effectively in clinical practice, it still needs to go through a significant number of clinical trials.

There are some limitations to our study. Firstly, this is a retrospective study with unavoidable selection bias. Secondly, after strict inclusion and exclusion criteria, our sample size was small, so a larger sample is needed to validate this study in the future. Thirdly, the NTIL group in this study has a large variety of diseases, and the value of using radiomics to help differential diagnosis of one of these diseases with TB will be discussed in the future. Fourthly, only CECT images scanned 55–60 s after contrast agent injection were selected in this study, the influence of different delayed scanning times on CECT's differential diagnostic ability can be further studied in the future. Fifthly, the use of multiple CT scanners in this study affected the reproducibility of the radiomics data. Although we used resampling for correction, we still need to use batch correction to minimize acquisition-related radiomics variability and verify the generality of the final model by stress testing in future studies. Finally, we used the rather time-consuming manual method of outlining ROIs. Compared with it, semi-automatic measurements are not only convenient and fast but also may have a better inter-observer agreement (36).

## Conclusions

In conclusion, radiomics helped to differentiate TB from NTIL presenting as solid pulmonary nodules or masses, and CECT may be a better choice. Combine RM we built obtained the best diagnostic efficacy and may outperform expert radiologists.

## Data availability statement

The raw data supporting the conclusions of this article will be made available by the authors, without undue reservation.

## Ethics statement

Approval was obtained from the Ethics Committee of the First Afliated Hospital of Dalian Medical University, the Ethics Committee of the Second Hospital of Dalian Medical University, and Dalian Public Health Clinical Center Ethics Committee. Written informed consent for participation was not required for this study in accordance with the national legislation and the institutional requirements.

## Author contributions

WZ: conceptualization, methodology, investigation, data curation, writing—original draft, and writing—review and editing. ZX: investigation, writing—original draft, and writing—review and editing. DT: validation, investigation, and data curation. KW, XL, and DQ: resources and data curation. MZ: software, visualization, and formal analysis. ZL: conceptualization, investigation, writing—review and editing, supervision, and project administration. All authors contributed to the article and approved the submitted version.

## Conflict of interest

Author MZ was employed by GE Healthcare. The remaining authors declare that the research was conducted in the absence of any commercial or financial relationships that could be construed as a potential conflict of interest.

## Publisher's note

All claims expressed in this article are solely those of the authors and do not necessarily represent those of their affiliated organizations, or those of the publisher, the editors and the reviewers. Any product that may be evaluated in this article, or claim that may be made by its manufacturer, is not guaranteed or endorsed by the publisher.

## Supplementary material

Supplementary Figure S1 Details of lesion selection for each patient.

Supplementary Figure S2 The modeling formulas of the NCECT and CECT RMs.

## Abbreviations

TB: tuberculosis
NTIL: non-tuberculous infectious lesions
NCECT: non-contrast-enhanced CT
CECT: contrast-enhanced CT
S1: apical segment of upper lobe
S2: posterior segment of upper lobe
S6: superior segment of lower lobe
IM: imaging model
RM: radiomics model
GBDT: gradient boosting decision tree
AMD: axis position maximum diameter
WBC: white blood cell
NEUT: neutrophil
LYMPH: lymphocyte
MONO: monocyte
Glu: glucose
ICC: inter- and intra-class correlation coefficients
ROC: receiver operating characteristic
AUC: area under the receiver operator characteristic curve
CI: confidence interval
H-L: Hosmer-Lemeshow
DCA: decision curve analysis
GLCM: gray-level co-occurrence matrix
GLDM: gray-level dependence matrix
GLRLM: gray-level run length matrix
GLSZM: gray-level size zone matrix
NGTDM: neighborhood gray-tone difference matrix.
