# Supplementary figures and images for: The adding value of contrast-enhanced CT radiomics: Differentiating tuberculosis from non-tuberculous infectious lesions presenting as solid pulmonary nodules or masses
Source: Front Public Health. 2022 Oct 4;10:1018527. doi: 10.3389/fpubh.2022.1018527 (PMC9577178; doi:10.3389/fpubh.2022.1018527)

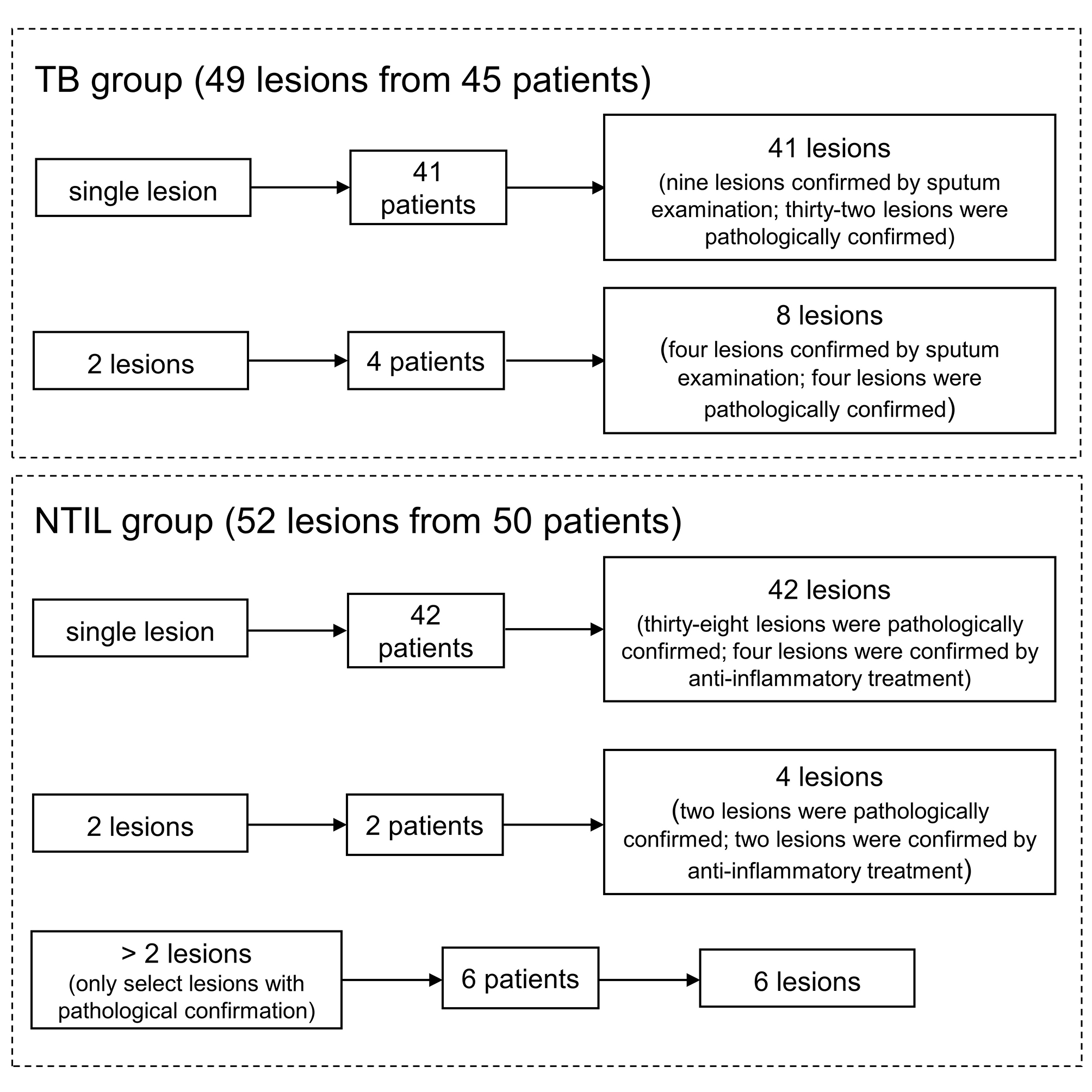

Supplement: Supplementary Figure S1 — Details of lesion selection for each patient. [file Image_1.JPEG]

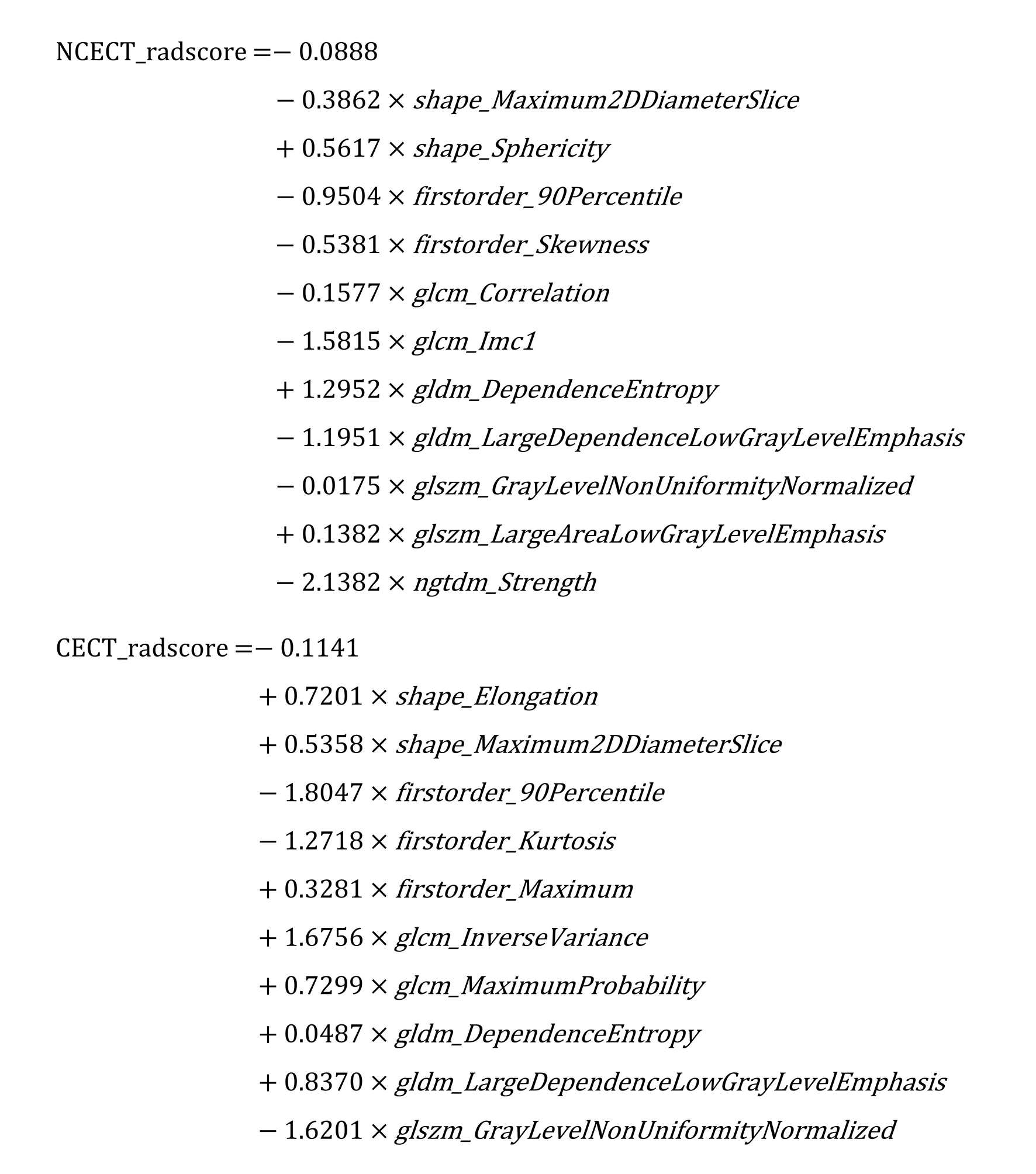

Supplement: Supplementary Figure S2 — The modeling formulas of the NCECT and CECT RMs. [file Image_2.JPEG]
